# Translation of a psychosocial education program for congenital adrenal hyperplasia in DVD format

**DOI:** 10.1186/1687-9856-2013-S1-P133

**Published:** 2013-10-03

**Authors:** Irene Mitchell, Tran Trieu Phuong Dong, Nguyen Bich Phuong, Nat Jackson, Pamela Dawes, Tien Nguyen

**Affiliations:** 1 Sydney Children's Hospital, Sydney, Australia; 2 Children's Hospital 2, Ho Chi Minh City, Vietnam; 3 Associate: CLAN Caring Living As Neighbours Australia; 4 Jacksonspeed Media Productions, Sydney, Australia; 5 South Sydney Health Interpreter Service, Sydney, Australia

## Introduction

Congenital Adrenal Hyperplasia (CAH) is a life threatening requiring good parental knowledge, in order to manage the daily care and any clinical problems arise. The impact of the condition is significant regardless of cultural background, with near death experiences and genital ambiguity occurring. Parents need to adjust to the burdens, that having a child with CAH creates, for all the family members. The authors have developed a validated CAH psychosocial education program (PEP) for families, which focuses on the essential information required to understand and manage CAH and which has been transformed into a DVD format, to improve access of families to educational resource information. This PEP has proven to be of vital importance in helping families manage this chronic condition[1]. However, it became apparent that families living form non-English speaking backgrounds, and those living in developing countries of South East Asia, did not have access to sufficient resource materials.

## Aim

This project aimed to translate the PEP in its Audiovisual format (DVD), into firstly Vietnamese, and then other languages. In doing so, its content could then be facilitated by one health professional.

## Method

A Medical Illustrations quote was obtained and funding was sourced. Five speaker presentations of the PEP were video-recorded live. Each was transcribed verbatim and edited in line with the slides. Transcripts were translated into Vietnamese by two medical professionals, and then narrated by a health services interpreter inline with the slide presentations. Discussion will include the process and the considerations required to develop such a comprehensive program in a translated format.

## Results

The DVD has been used for newly diagnosed families at our centre, and in country NSW. As expertise with this condition is limited only to specialist centres, the DVD has since been sourced by the CAH Support Group Australia (CAHSGA), and internationally by CLAN, (Caring Living as Neighbours) an organisation providing healthcare needs for resource poor countries. Having completed the Vietnamese translation, we are currently pursuing an Indonesian translation.

## Conclusion

Developing comprehensive education programs in a DVD format is necessary for supporting families in caring for their child. Ensuring families of non-English speaking backgrounds receive necessary and appropriate information to enable them to manage their child’s condition is the responsibility of all health professionals.
